# Enzymatic Conversion of Oleuropein to Hydroxytyrosol Using Immobilized *β*-Glucosidase on Porous Carbon Cuboids

**DOI:** 10.3390/nano9081166

**Published:** 2019-08-14

**Authors:** Alexandra V. Chatzikonstantinou, Elena Gkantzou, Eleni Thomou, Nikolaos Chalmpes, Kyriaki-Marina Lyra, Vasiliki G. Kontogianni, Konstantinos Spyrou, Michaela Patila, Dimitrios Gournis, Haralambos Stamatis

**Affiliations:** 1 Biotechnology Laboratory, Department of Biological Applications and Technologies, University of Ioannina, 45110 Ioannina, Greece; 2 Department of Materials Science & Engineering, University of Ioannina, 45110 Ioannina, Greece; 3 Section of Organic Chemistry & Biochemistry, Department of Chemistry, University of Ioannina, 45110 Ioannina, Greece

**Keywords:** *β*-glucosidase, carbon cuboids, hydroxytyrosol, oleuropein, bio-catalysis, nano-biocatalyst

## Abstract

In the present study, we developed novel *β*-glucosidase-based nano-biocatalysts for the bioconversion of oleuropein to hydroxytyrosol. Using non-covalent or covalent immobilization approaches, *β*-glucosidases from almonds and *Thermotoga maritima* were attached for the first time on oxidized and non-oxidized porous carbon cuboids (PCC). Various methods were used for the characterization of the bio-nanoconjugates, such as Fourier transform infrared spectroscopy (FTIR), X-ray photoelectron spectroscopy (XPS), atomic force microscopy (AFM), and fluorescence spectroscopy. The oxidation state of the nanο-support and the immobilization procedure play a key role for the immobilization efficiency or the catalytic activity of the immobilized *β*-glucosidases. The nano-biocatalysts were successfully used for the hydrolysis of oleuropein, which leads to the formation of its bioactive derivative, hydroxytyrosol (up to 2.4 g L^−1^), which is a phenolic compound with numerous health benefits. The bio-nanoconjugates exhibited high thermal and operational stability (up to 240 h of repeated use), which indicated that they are efficient tools for various bio-transformations.

## 1. Introduction

Bio-catalysis has rapidly gained ground in almost every catalytic process due to its advantages, such as selectivity (region-, chemo-, stereo-) and low environmental impact when compared to traditional synthetic methodologies. Heterogeneous bio-catalysis refers to enzymes in a water-insoluble form and is preferred lately for industrial production processes, mainly for the easy separation of the products, but also for the ability to reuse the biocatalyst for multiple reaction cycles [1]. Heterogeneous biocatalysts are enzymes immobilized in various solid materials such as polymers, silica, nanomaterials, etc. [2].

The benefits of enzyme immobilization, along with using nanomaterials as matrices, have gained interest during the last decade [3]. Carbon-based nanomaterials have dominated the world of nano-immobilization, since they combine both effectiveness and biocompatibility [4,5]. Researchers have managed a wide range of carbon nanomaterials, regarding both shapes and sizes, with each one competing each other for its benefiting effect on enzymatic performance. It is well known, that the structural characteristics of the nanomaterials, such as size, shape, porosity, and surface chemistry, can affect the immobilization and the catalytic behavior of the immobilized enzyme. Carbon porous nanomaterials have long attracted the attention of enzyme supports, due to their important features, regarding enzyme immobilization, such as the increased surface area and the pore volume they provide in order to achieve higher protein loading, while they exhibit no spatial restrictions upon enzyme molecules [6,7,8]. Porosity also seems to influence the activity of the biocatalyst since it can facilitate substrate accessibility to the enzyme [9,10]. Functionalized carbon-based nanomaterials also excel for their unique properties that functional groups provide them with. Different kinds of functionalization modify the properties of the nanomaterials, which affects their interaction with proteins, and, thus, the catalytic characteristics of the immobilized enzymes [11,12]. Several studies have shown that the functionalization of carbon-based nanomaterials enhances not only the enzyme loading and the catalytic activity, but also the operational and thermal stability of the biocatalysts [13,14].

A brand-new member of the carbon family, which combines porosity and capability to accept functional groups on its surface, has recently been reported as a high performance material [15]. This novel nanostructure is called porous carbon cuboids (PCCs) and combines a series of intriguing properties, such as light weight, unusual ultra-hydrophilic behavior, great stability, surface heterogeneity, and a very high hierarchical porosity (estimated approximately 800–900 m^2^ gr^−1^) [15]. In contrast with graphene, PCC do not need to pass through the oxidation state due to the high number of functional groups they possess on the surface, which renders them highly hydrophilic. PCC show a significant number of N:C and O:C active sites, which, in combination with the narrow micropore size distribution, constitute a very promising sorbent. The advantage that they present over other carbon materials is the fact that they are stable up to 400 °C, whereas most of them cannot be functional in temperatures further than 300 °C. By further treatment of the PCCs with strong oxidizing agents (employing a modified Staudenmaier’s method), multiple oxygen functionalities (such as carboxyl, hydroxyl, and epoxy) can be introduced, which increases the number of active sites [16,17,18,19,20]. These oxidized PCCs, as well as the pristine ones, have the ability to interact with bacteria and algae, as recently shown [16].

**Figure 1:**
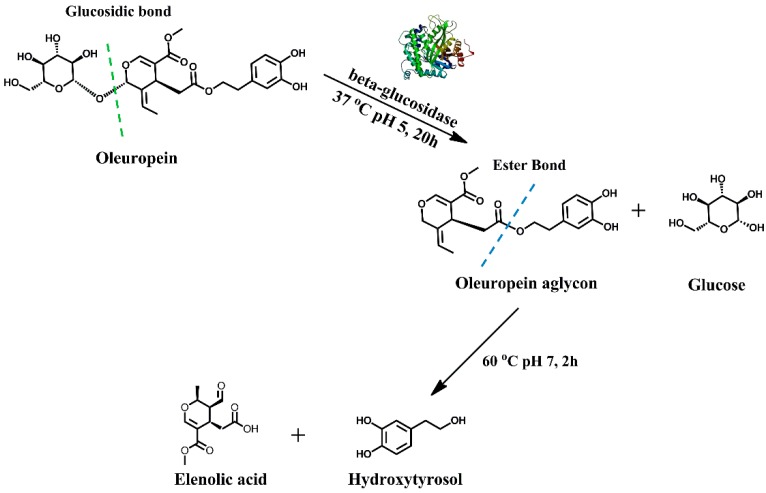
Transformation of oleuropein to hydroxytyrosol.

In the present study, pristine PCCs and surface oxidized (with -COOH and -OH groups) analogues (PCCox) were used, for the first time, as nano-supports for the covalent and non-covalent immobilization of two *β*-glucosidases (from almond and *Thermotoga maritima*) that are widely used in various biocatalytic processes with industrial interest [21,22]. These novel nano-biocatalysts were tested for their ability to catalyze the bioconversion of oleuropein (OLE) to hydroxytyrosol [3,4-dihydroxyphenylethanol (HT)] (Figure 1). OLE is a hydrophilic phenolic antioxidant found in all parts of the olive tree, with high concentrations in the dry leaf extract and also abundant in olive mill wastewaters. HT is the main degradation product of OLE, which is considered to be one of the strongest antioxidants in nature [23] with superior biological activities than the parent compound OLE [24,25]. HT is used as a food supplement and is used in the cosmetic industry. In addition, it is considered to be a potent drug agent and a food additive due to its anti-inflammatory, anticarcinogenic, neuroprotective, and antiapoptotic activity [23]. However, HT is found in low concentration in nature, has low extraction yields from natural sources, and is difficult to chemically synthesize. Therefore, the production of pure HT is a high cost procedure [26]. Furthermore, the development of biotechnological approaches for the synthesis of HT is of great interest. Τhe synthesis of HT through the conversion of OLE from olive leaves or olive mill wastewaters could be an optimal direction for a cost-effective production of HT [23]. Various hydrolytic enzymes have been employed for the conversion of OLE to HT such as esterases, lipases, xylanases, cellulases, and hemicellulases [27]. Natural or recombinant *β*-glucosidases have been proved to be the key enzymes for OLE hydrolysis and have been used either as free or immobilized enzymes [27,28,29,30,31].

A combination of spectroscopic, microscopic, and biochemical techniques was applied for the characterization of these novel bio-nanoconjugates with respect to their catalytic behavior. Moreover, the effect of the surface chemistry of PCCs and the immobilization approaches that were used on the catalytic activity as well as thermal and operational stability of the immobilized enzymes was investigated.

## 2. Materials and Methods

### 2.1. Materials

*β*-Glucosidase from almonds (Albgl) 9.5 U mg^−1^ was purchased from Sigma-Aldrich (St. Louis, MO, USA) and was used with no further purification. N′-ethylcarbodiimide hydrochloride (EDC), N-Hydroxysuccinimide (NHS), *p*-nitrophenol (*p*-NP), and *4*-Nitrophenyl *β*-*D*-glucopyranoside (*p*-NPG) were purchased from Sigma-Aldrich (St. Louis, MO, USA), *β*-glucosidase from *Thermotoga maritima* (Tmbgl) 85 U mg^−1^ was purchased from Megazyme (Chicago, IL, USA) and was used with no further purification. Oleuropein and hydroxytyrosol were purchased from Extrasynthese (Lyon, France). 4,4′-Bipyridine (98%) and Potassium chlorate (KClO_3_, 99+%) were purchased from Alfa-Aesar (Kandel, Germany). Ethanol (absolute, for analysis) was purchased from Merck (KGaA Darmstadt, Germany). Nitric acid (HNO_3_, 65%) was purchased from Chem-Lab (Zedelgem, Belgium), and Sulphuric acid (H_2_SO_4_, 96%) was purchased from Panreac (Castellar del Vallès, Spain).

### 2.2. Synthesis of Porous Carbon Cuboids (PCC) and Oxidized PPC (PCCox)

The same procedure that has been described in our previous work was followed for the synthesis of PCC [16]. Two solutions were prepared. The first one consisted of 1 g of Pluronic dissolved in 100 mL of 0.1 M 4,4′-bipyridine in water−ethanol (volume ratio of 1:17) and the second one was an aqueous solution of copper (II) chloride (900 mL, 5.6 mM CuCl_2_·2H_2_O). The first solution was then poured rapidly into the second one under vigorous stirring. The formed products were retrieved through centrifugation, washed three times with water, and air dried. The product was then pyrolyzed under argon flow (500 °C for 2 h) and the copper species were leached away (stirred in a 4 M HNO_3_ aqueous solution for 24 h). The final black powder was collected by air drying after being washed with water until pH was ~5.0.

Likewise, the PCC was treated with strong oxidizing agents by employing a modified Staudenmaier’s method, in order to introduce more active sites (PCCox) [16,17,18,19,20]. Additionally, 70 mg of PCC were added to a mixture of H_2_SO_4_ and HNO_3_ (1.4 mL and 2.8 mL, respectively) while keeping the temperature at ~0 °C in an ice-water bath. Under constant stirring and cooling, small quantities of KClO_3_ (0.7 g in total) were added slowly to the mixture. The reaction was terminated after 18 h by pouring the mixture into distilled water and the final product was washed until pH∼6.0 and was dried at room temperature.

### 2.3. Covalent Immobilization of β-Glucosidase on PCC and PCCox

Albgl and Tmbgl were covalently immobilized on PCC and PCCox via cross-linking agents such as EDC and NHS, which can link the -COOH and -OH groups of nanomaterials with the free amino groups of the enzyme [32,33]. In a typical procedure, 4 mg of nanomaterial were dispersed in 5 mL H_2_O and 1 mL HEPES (*N*-(2-Hydroxyethyl) piperazine-*N*′-(2-ethanesulfonic acid)) solution (pH 7.0, 50 mM) in an ultrasonic bath for 30 min. After the dispersion, 2.3 mL of a 50 mg mL^−1^ NHS aqueous solution and 1.2 mL of a 10 mg mL^−1^ EDC aqueous solution were added and the mixture was incubated under stirring for 30 min at 30 °C. The modified nanomaterial was then separated by centrifugation at 4000 rpm for 10 min and washed with HEPES solution in triplicate to remove the excess of EDC and NHS. The activated nanomaterial was re-dispersed in 6 mL HEPES solution 50 mM at the optimum pH values (pH 5.0 and pH 6.5 for Albgl and Tmbgl, respectively). Then, 0.3 mg (estimated by Bradford assay) was added and the mixture was incubated under stirring at 30 °C for 1 h. The bio-nanoconjugates were separated by centrifugation at 4000 rpm for 10 min and washed with the proper HEPES solution in triplicate to remove the excess of enzyme and then left to dry in silica at 4 °C. The nano-biocatalysts were labeled as PCC-Albgl-cov, PCCox-Albgl-cov, PCC-Tmbgl-cov, and PCCox-Tmbgl-cov.

### 2.4. Non-Covalent Immobilization of β-Glucosidase on PCC and PCCox

Albgl and Tmbgl were attached via physical absorption on PCC and PCCox. Additionally, 4 mg of nanomaterial were dispersed in 6 mL HEPES solution 50 mM, at the optimum pH values (pH 5.0 and pH 6.5 for Albgl and Tmbgl, respectively), in an ultrasonic bath for 30 min. Furthermore, 0.3 mg of *β*-glucosidase were added and the mixture was incubated under stirring at 30 °C for 1 h. The bio-nanoconjugates were separated by centrifugation at 4000 rpm for 10 min and washed with the proper HEPES solution in triplicate to remove the excess of the enzyme and then left to dry in silica at 4 °C. The nano-biocatalysts were labeled as PCC-Albgl-nc, PCCox-Albgl-nc, PCC-Tmbgl-nc, and PCCox-Tmbgl- nc.

### 2.5. Determination of the Immobilization Yield

The Bradford assay was used to determine the immobilization yield by estimating the amount of the protein in the supernatant before and after the immobilization [34]. The amount of immobilized *β*-glucosidase on PCC and PCCox was calculated as the difference of the protein concentration in the supernatant before and after the immobilization. All experiments were carried out in duplicate.

### 2.6. Determination of the Hydrolyitc Activity of Immobilized β-Glucosidase

The hydrolytic activity of *β*-glucosidases was determined spectrophotometrically by estimating the *p*-NP released from the hydrolysis of *p*-NPG at 410 nm, as described before [35]. The catalytic assay was carried out in citrate-phosphate buffer solution 100 mM, pH 5.0, and pH 6.5 for Albgl and Tmbgl, respectively, at 50 °C for 10 min. The reaction was started by adding an appropriate amount of immobilized *β*-glucosidase (0.1 or 0.01 mg mL^−1^) in a *p*-NPG solution (2 mM). The reaction was stopped by adding 0.5 mL of a 10% w/v Na_2_CO_3_ solution and the reaction product (*p*-NP) was measured at 410 nm. The amount of the released *p-*NP was quantified using a *p*-NP standard curve. One *β*-glucosidase unit (U) was defined as the µmol min^−1^ of *p*-NP that results from 1 mg of immobilized enzyme under the above-defined conditions.

### 2.7. Thermal Stability of Free and Immobilized β-Glucosidases

Thermal stability studies of *β*-glucosidases were performed by incubating free or immobilized enzymes in citrate-phosphate buffer 100 mM, pH 5.0, and pH 6.5 for Albgl and Tmbgl, respectively, at 60 °C for 24 h. The amount of the enzyme was 0.01 mg mL^−1^ or 0.01 µL mL^−1^ of free Albgl and Tmbgl, respectively, and 1 mg mL^−1^ of immobilized *β*-glucosidase, in all cases. The remaining hydrolytic activity of *β*-glucosidase was determined, at different time intervals, through the hydrolysis of *p*-NPG, as described before. All experiments were performed in triplicate.

### 2.8. Hydrolysis of Oleuropein to Hydroxytyrosol

The enzymatic hydrolysis of OLE was performed in citrate-phosphate buffer 100 mM, pH 5.0, and pH 6.5 for Albgl and Tmbgl, respectively. In addition, 1 mg of a nano-biocatalyst was dispersed in 2 mg mL^−1^ solution of OLE (3.7 mM), which was followed by 24 h incubation at 37 °C under shaking (750 rpm). After the enzymatic hydrolysis of OLE (step 1) (Figure 1), a second non-enzymatic step followed to give rise to HT. In step 2, the reaction mixture was incubated at 60 °C, pH 7.0 for 2 h under shaking (750 rpm).

### 2.9. High Performance Liquid Chromatography (HPLC) Analysis

The identification and quantification of OLE and HT were performed by high performance liquid chromatography (HPLC) (Shimadzu, Tokyo, Japan) using a μBondapack C18 column, particle size 10 μm, length 300 mm, diameter 3.9 mm, and a diode array UV detector. The mobile phase was acetonitrile (A) and 0.1% acetic acid in water (B) of 20–80%. The elution conditions applied were: 0–30 min, 50–50% (v/v) A:B, 30–35 min, 50–50% A:B and 35–40 min, and 20–80% A:B. The elution was performed at 27 °C with a flow rate of 1 mL min^−1^ and the samples were detected at 280 nm. The quantification/characterization of OLE and HT was based on standard samples and calibration curves at the same conditions.

### 2.10. Reusability Studies of Immobilized β-Glucosidases

The reusability of immobilized *β*-glucosidases was estimated by performing consecutive operating cycles of the hydrolysis of OLE, as described before. Each reaction cycle was carried out for 24 h at 37 °C under shaking (750 rpm). After each run, the immobilized enzyme was separated from the reaction mixture with centrifugation and washed thoroughly with citrate-phosphate buffer solution 100 mM, pH 5.0, and pH 6.5 for Albgl and Tmbgl, respectively, and then it was re-used. All experiments were performed in triplicate.

### 2.11. Fourier-Transform Infrared Spectroscopy (FTIR)

An FTIR-8400 infrared spectrometer (Shimadzu, Tokyo, Japan) equipped with a deuterated triglycine sulfate (DTGS) detector was used for the Fourier-transform infrared spectroscopy (FTIR) analysis. All spectra were recorded in the range of 400 to 4000 cm^−1^ and were an average of 32 scans. All samples were in the form of KBr pellets containing ca. 2 wt% of the enzyme, the nanomaterials (PCC and PCCox), or the nano-biocatalysts. Equation (1) was used to calculate the correlation coefficient *r* in order to determine the similarity between two FTIR spectra [36,37,38,39].

(1) $$r=\sum x_{i}y_{i}/\sqrt{\sum x_{i}^{2}\sum y_{i}^{2}},$$

where *x* and *y* are the spectral absorbance values of free and immobilized enzyme, respectively, at the *i*th frequency position. For the calculation, the absorbance values of the spectra after smoothing in the region of 1600 to 1700 cm^−1^ (amide region I) were used. The correlation coefficient *r* value will be equal to one (*r* = 1) for identical spectra.

### 2.12. Fluorescence Spectroscopy

A luminescence spectrofluorometer Jasco-8300 (Tokyo, Japan) was used for all fluorescence measurements using a solid sample holder. Immobilized *β*-glucosidase was used in aqueous solutions at concentration of 1 mg mL^−1^, while free Albgl and Tmbgl at concentration of 0.13 mg mL^−1^. Samples were deposited onto silicon wafers (P/Bor, single side polished) from aqueous solutions by drop casting. The fluorescence emission spectra were recorded from 300 to 400 nm after exciting at 280 nm, with a scan speed of 100 nm min^−1^ at room temperature. Slit widths with a nominal band pass of 5 nm were used for both excitation and emission ray.

### 2.13. X-ray Photoelectron Spectroscopy

X-ray photoelectron spectroscopy (XPS) measurements were performed in ultra-high vacuum at a base pressure of 2 × 10^−10^ mbar with a SPECS GmbH spectrometer equipped with a monochromatic MgKa source (hv = 1253.6 eV) and a Phoibos-100 hemispherical analyzer (Berlin, Germany). The spectra were collected in normal emission and energy resolution was set to 1.16 eV to minimize measuring time. All binding energies were referenced to the C1s core level at 284.6 eV [40]. Spectral analysis included a Shirley background subtraction and a peak deconvolution employing mixed Gaussian-Lorentzian functions, in a least square curve-fitting program (WinSpec) developed at the Laboratoire Interdisciplinaire de Spectroscopie Electronique, University of Namur, Belgium.

### 2.14. Atomic Force Microscopy

Atomic force microscopy (AFM) images were collected in tapping mode with a Bruker Multimode 3D Nanoscope (Ted Pella Inc., Redding, CA, USA), using a microfabricated silicon cantilever type TAP-300G, with a tip radius <10 nm and a force constant of ~20–75 N m^−1^.

### 2.15. Raman Spectroscopy

Raman spectra were collected with a Micro-Raman system RM1000 RENISHAW (RENISHAW, Old Town, UK) using a laser excitation line at 532 nm (laser diode) in the range of 1100 to 1800 cm^−1^. Raman scattering was collected by means of an optical microscope equipped with 50× and 100× lenses.

## 3. Results and Discussion

In the present work, we developed robust nano-biocatalysts as efficient tools in various biocatalytic processes with industrial interest. More specific, we investigated the immobilization of *β*-glucosidases on PCCs and functionalized PCCs with -COOH and -OH groups. The enzymes were immobilized with both covalent and non-covalent approaches. Covalent immobilization was carried out through EDC/NHS linkers while non-covalent immobilization was performed through physical adsorption. This way, we could investigate how the immobilization approach or the surface chemistry of the nanomaterial affects the immobilization efficiency, as well as the catalytic behavior of the immobilized *β*-glucosidases.

### 3.1. Characterization of PCC and PCCox

**Figure 2:**
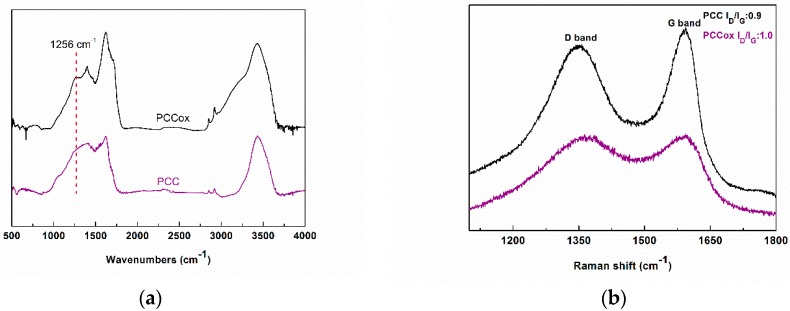
(**a**) Fourier-transform infrared spectroscopy (FTIR) spectra of the porous carbon cuboids (PCC) and oxidized porous carbon cuboids (PCCox). (**b**) Raman spectra of the PCC and PCCox.

Raman, FTIR, and XPS spectroscopy were employed to characterize both the pristine and oxidized PCC. The infrared spectra of PCC and PCCox are depicted in Figure 2a. In the infrared (IR) spectra of the PCC, the peaks between 2843 and 2931 cm^−1^ are due to the stretching vibrations of CH_2_ groups, while the broad band centered to 3431 cm^−1^ is attributed to the adsorbed H_2_O deformation, which is indicative of the hydrophilic nature of the carbon material. The weak band of the hydroxyl stretching vibrations of the C-OH groups, expected around 3550 cm^−1^ [41], is not visible since it is superimposed in this broad. Moreover, the band at 1402 cm^−1^ is attributed to the aromatic C=C functional group, while the main peak at 1623 cm^−1^ is due to C=O stretching vibrations of the carboxyl groups [42]. The latter is more pronounced and intense in the case of PCCox spectrum due to a higher degree of oxidation. In the same way, the band at 1256 cm^−1^, due to vibrations of the epoxy groups (COC) [43,44,45], is more distinct in the PCCox sample, compared to the pristine one, as a result of the extensive oxidation process.

The Raman spectra of the porous carbon cuboids before and after oxidation are shown in Figure 2b. The two characteristic graphitic peaks, at 1350–1370 and 1593 cm^−1^ corresponding to graphitic D and G bands, respectively, were observed in both Raman spectra of the synthesized nanomaterials. The D band is associated with an *sp*^3^-hybridized carbon, whereas the G-band is associated with an *sp*^2^-hybridized carbon atoms. The intensities ratio I_D_/I_G_ express the degree of disorder of the carbon lattice. After oxidation, the ratio was lightly increased to 1.0 (from 0.9 in the pristine PCC), which indicates a change in hybridization of the carbon atoms from *sp*^2^ to *sp*^3^ due to the increase of the population of the oxygen-containing groups, as the result of the oxidation process [46].

The successful oxidation of the PCC was further supported by XPS spectroscopy. The results of pristine PCC and oxidized PCC (PCCox) have been reported in our previous work [16]. According to this work, pristine PCC possess oxygen functionalities, apart from the main C-C frame, such as C-O, C=O, and C(O)O. After oxidation, a significant decrease to the C-C bond (from 51.6% for PCC to 34% for PCCox) can be deduced from the insertion of multiple oxygen moieties to the main body of PCC. Most of the oxygen functionalities increased in ratio (such as C-OH and C(O)O), while the insertion of an additional peak is observed, derived from the epoxy groups due to oxidation treatment.

**Figure 3:**
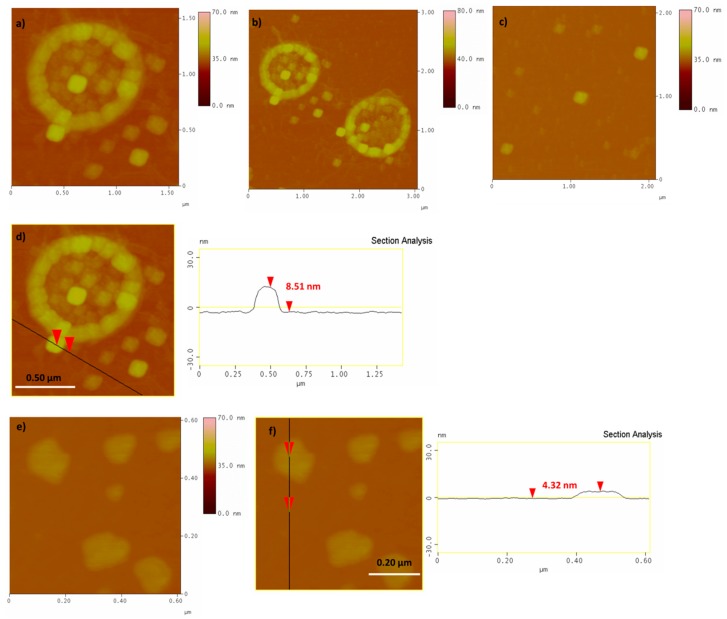
Atomic force microscopy (AFM) height images and cross section analysis of PCC (**a**–**d** images) and PCCox (**e**,**f** images).

Representative AFM images of the PCC and PCCox are presented in Figure 3, which confirms the formation of the cubic structure. In addition, the topographic image of an isolated cuboid, as revealed from the cross-sectional analysis, is observed in Figure 3d with an average thickness of 8.51 nm. Accordingly, from the AFM images of PCCox, it is clearly observed that the oxidation process influenced the cubic shape of the carbon nanostructures, as well as the thickness of the PCCox, which is decreased, as shown in Figure 3f. All these values are in agreement with our previous work [16]. It is interesting to note that, while isolated cubes are observed frequently in the surface of the Si-wafer, there are areas where PCCs tend to get organized into ring structures, by integrating monomeric cuboids (Figure 3a,b,d). This behavior could be due to intramolecular interactions between adjacent cuboid nanoparticles in water, where the formation of hydrogen bonding (via carboxyl and/or hydroxyl groups) occurs between neighboring cuboids, which results in the formation of a 2D ring-like supramolecular structure on the surface of a silicon wafer. In fact, when pH was raised to 11.0 (from 5.6 that was initially), due to deprotonation of the carboxyl (or hydroxyl) groups, intramolecular hydrogen bonding was no longer possible and no ordered structure was observed on the surface. The presence of water seems to be crucial for the formation of these ring-like structures, since, when experiments were performed in ethanol (instead of water), no similar ring-like formations were observed. On the other hand, oxidized PCCs, due to the greater number of oxygen functionalities on their surface, are more hydrophilic than pristine PCCs and prefer to be dispersible and isolated in the aqueous medium. Thus, upon deposition, the majority do not tend to aggregate into ring structures (Figure 3e,f).

### 3.2. Immobilization Efficiency and Activity of Immobilized β-Glucosidase

In the present work, PCCs and functionalized PCCs with multiple oxygen functionalities (PCCox) were used, for the first time, as nano-supports for enzyme immobilization. It is well known that the functionalization of carbon-based nanomaterials plays a key role in the development of interactions between the nanomaterial and the enzyme molecules, which affects the catalytic behavior of the latter [4,12,47]. The immobilization of Albgl and Tmbgl was carried out through physical adsorption and covalent immobilization. Physical adsorption of the enzyme onto carbon-based nanomaterials is based on hydrophobic interactions, electrostatic and van der Walls forces, and hydrogen bonding [12,48]. Covalent attachment of the enzyme onto the -OH and -COOH groups of these nanomaterials was carried out by using EDC and NHS as cross-linkers. In this case, stable amide bonds are formed between the enzyme and the nanomaterial [48], even though the co-existence of physical adsorption of the enzyme cannot be excluded.

Table 1 presents the immobilization yield of Albgl and Tmbgl on PCC and PCCox. In all cases, the mass ration of the enzyme to the nanomaterial was 0.1. Tmbgl was successfully immobilized, for the first time onto nanomaterials. The immobilization efficiency of Tmbgl does not seem to depend on the immobilization procedure or the nano-support. It should be noted that, in a range of pH 5–7, no significant differences in the immobilization yields were observed. As it can be seen in Table 1, high immobilization yields are achieved (>90%) in all cases. *β*-Glucosidase from *Thermotoga maritima* was previously successfully immobilized on chitin beads and was used for the hydrolysis of lactose [49]. On the other hand, the immobilization of Albgl on PCC and PCCox is also successful but the immobilization yield seems to depend on the immobilization procedure and the nano-support that was used. The highest immobilization yield (90%) is observed when PCCox is used as a nano-support for the covalent immobilization of the enzyme, which points out the importance of the functionalization of PCCs. Previous works have shown that covalent immobilization on surface O-functionalities can increase the enzyme loading [11,50]. As far as it concerns non-covalent immobilization, a higher immobilization yield is obtained in the case of PCCs. The presence of oxygen functional groups can lead to lower immobilization yields on carbon-based nanomaterials, as reported elsewhere [47]. Similar, and, in some cases, lower, immobilization efficiencies have been previously reported for *β*-glucosidase immobilized on various nanomaterials, such as single-walled and multi-walled carbon nanotubes and hybrid magnetic graphene oxide nanoparticles [35,51,52].

The hydrolytic activity of the bio-nanoconjugates was investigated and the initial reaction rates are presented in Table 1. The catalytic activity of free *β*-glucosidases is higher compared to the immobilized enzymes. This reduction in activity is in accordance with that previously reported for *β*-glucosidase and other enzymes, and could be attributed either to conformational changes in the enzyme molecule upon immobilization or to mass transfer effects, which can reduce the catalytic efficiency of the immobilized enzyme [35,53]. It is interesting to note that the catalytic activity of the immobilized *β*-glucosidase does not seem to be correlated with the enzyme loading, but it seems to depend on the nature of the nano-support. As can be seen in Table 1 when PCCox is used as a nano-support, higher hydrolytic activity is achieved in all cases. The reduced activity that is observed in the case of PCCs could be attributed to the interaction of enzymes with the hydrophobic surface of the nanomaterials, which could lead to undesirable conformational changes in the protein molecules, and, hence, loss of their catalytic activity. Similar behavior was observed for the immobilization of lipases on carbon nanotubes and graphene-based nanomaterials [4]. Moreover, it is expected that a significant part of the enzyme molecules is mainly adsorbed in the macro-pores of the PCCs, which causes restricted diffusion of the substrate on the active sites of immobilized enzyme molecules [6]. On the other hand, the oxidation of PCC is expected to reduce the porosity of the nanomaterials, which prevents the adsorption of the enzymes on the pores of the nanomaterials. Furthermore, it is expected that the enzymes are attached to the oxygen functional groups of the PCCox nanomaterials. The presence of these oxygen functional groups could create a more hydrophilic microenvironment around immobilized enzyme molecules that facilitates the diffusion of the substrate to the active site of the enzyme, and, thus, increases its catalytic activity [54].

### 3.3. Characterization of Bio-Nanoconjugates

**Figure 4:**
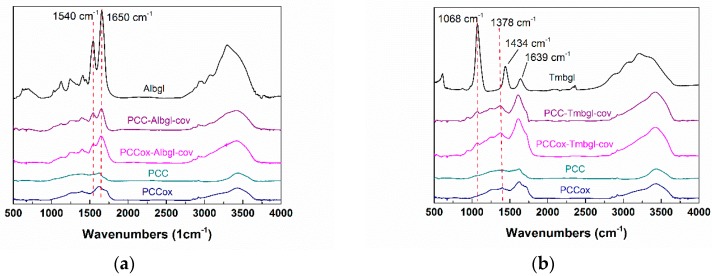
FTIR spectra of (**a**) PCC-Albgl-cov and PCCox-Albgl-cov compared to those of free Albgl and bulk PCC and PCCox. (**b**) PCC-Tmbgl-cov and PCCox-Tmbgl-cov compared to those of Tmbgl and bulk PCC and PCCox.

To confirm the presence of *β*-glucosidase on PCC and PCCox, FTIR spectroscopy was used. Figure 4 illustrates the FTIR spectra of free and immobilized Albgl and Tmbgl. The spectrum of free Albgl shows a typical protein spectrum that presents two absorption bands at 1540 cm^−1^ and 1650 cm^−1^ [55], associated with the amide bond due to the C=O stretching vibrations, and is directly related to the backbone conformation. Immobilized Albgl also depicts two bands at 1540 cm^−1^ and 1650 cm^−1^, which suggests the successful conjugation of the enzyme onto the PCC and PCCox. Similarly, the spectrum of free Tmbgl presents three absorption bands at 1378 cm^−1^, 1343 cm^−1^, and 1639 cm^−1^ associated with the amide bond region. Immobilized Tmbgl depicts a band at 1068 cm^−1^ and a band at 1378 cm^−1^, which is slightly transposed when compared to the free enzyme. These peaks indicate the successful immobilization of the enzyme onto the PCC and PCCox. FTIR analysis was also employed to investigate the conformational changes of the enzyme upon immobilization onto PCC and PCCox compared to the structure of the free enzyme. To evaluate the differences between the FTIR spectra of free and immobilized *β*-glucosidase, the correlation coefficient, *r*, was estimated [38,39,56]. According to the results, the correlation coefficient r for immobilized Albgl and Tmbgl is 0.99 and 0.98, respectively, which indicates that the structure of the immobilized enzyme is not significantly altered.

**Figure 5:**
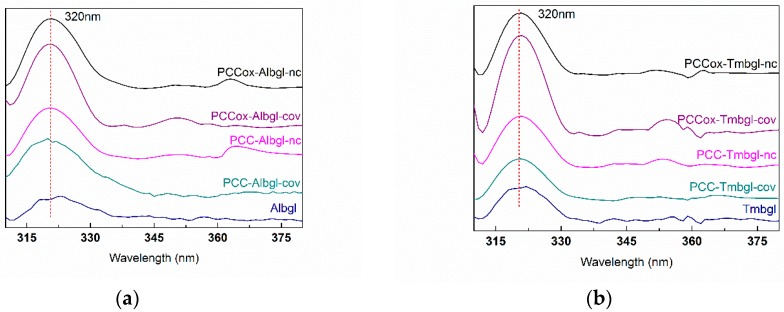
Fluorescence spectra of (**a**) free and immobilized Albgl on PCC and PCCox. (**b**) Free and immobilized Tmbgl on PCC and PCCox.

Fluorescence spectroscopy has been used before to confirm the presence of the enzyme on solid material [57,58]. Herein, the fluorescence emission spectra of free and immobilized enzymes were recorded in order to further confirm the presence of *β*-glucosidase on PCC and PCCox (Figure 5). Excitation was set at 280 nm and the emission intensity was recorded in the range of 300 to 400 nm. The maximum emission wavelength of free *β*-glucosidase was found at 320 nm for both Albgl and Tmbgl. The fluorescence spectra of bulk nanomaterials PCC and PCCox were also recorded at the same conditions and no emission was observed in the range of 300 to 400 nm. As can be seen in Figure 5, in all cases, the fluorescence spectra of bio-nanoconjugates reveal a maximum emission at 320 nm, which indicates the presence of *β*-glucosidase and, therefore, the successful immobilization of the enzyme.

**Figure 6:**
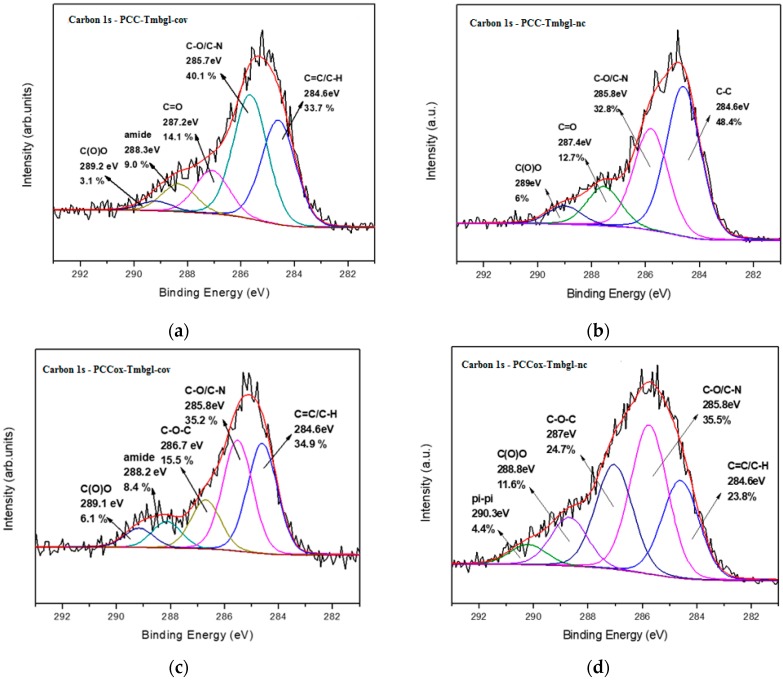
C1s photoelectron spectra of (**a**) PCC-Tmbgl-cov, (**b**) PCC-Tmbgl-nc, (**c**) PCCox-Tmbgl-cov, and (**d**) PCCox-Tmbgl-nc.

X-ray photoelectron spectroscopy of bio-nanoconjugates was employed to the porous cuboids and porous cuboids after the oxidation process in order to distinguish the type of interaction (covalent or non-covalent) between the enzymes and the PCC matrix. From the carbon 1s photoelectron spectra of the PCC-Tmbgl-cov (Figure 6a), five contributions are deduced from the C=C/C-H bonds at 284.6 eV (33.7%), the C-O/C-N bonds from the C-O functional groups of PCC, and the C-N and C-O bonds from the enzyme representing 40.1% of the whole carbon spectra. At 287.2 eV, the contribution of C=O bonds (14.1%) is detected, while a very unique peak that attests to the successful covalent bonding between the Tmbgl and PCC is observed at 288.3 eV due to the creation of the amide groups [59] representing 9.0% of the carbon amount. Lastly, a last fitted peak at 289.2 eV is due to the carboxyl groups. This peak represents just 3.1% and this is because carboxyl groups participate in the covalent bond to create amide groups. Accordingly, from the carbon 1s photoelectron spectra of PCC-Tmbgl-nc (Figure 6b), four contributions are displayed due to C=C/C-H located at 284.6 eV covering 48.4% of the carbon spectra, C-O/C-N groups centered at 285.8 eV (32.8%), carbonyl groups at 287.4 eV (12.7%), and, lastly, a last fitted peak at 289.0 eV due to carboxyl groups representing 6.0% of the whole carbon amount. No amide peak is observed, while the carboxyl group peak is much higher than in the case of PCC-Tmbgl-cov, which is a sign of non-covalent interaction between the enzyme and the PCC nanomaterials.

XPS spectra of PCCox-Tmbgl-cov are displayed in Figure 6c. Fitting on the C1s photoelectron peak reveals five different contributions derived from C=C/C-N at 284.6 eV (34.9%), C-O/C-N at 285.8 eV (35.2%), and a contributed peak derived from epoxy groups at 286.7 eV due to the initial oxidation treatment of the PCC representing 15.5% of the carbon spectra. The amide groups due to the covalent interaction of Tmbgl and the carboxyl groups of the PCC are centered to 288.2 eV (8.4%), while some unaffected carboxyl moieties are observed at 289.4 eV (6.1%). Lastly, for PCCox-Tmbgl-nc (Figure 6d), fitted peaks from C=C/C-H at 284.6 eV (23.8%), C-O/C-N at 285.8 eV (35.5%), and epoxy groups at 287.0 eV (24.7%) are observed. There is no contributed peak from amide bonds, which reveals the non-covalent nature of the interaction between the enzyme and the PCC nanostructure. A peak at 289.0 eV is due to carboxyl groups and represents 11.6% of the whole carbon contribution. Compared to PCCox-Tmbgl-cov, the contribution from carboxyl groups is higher because the enzyme does not interact covalently with them. A last fitted peak at 290.3 eV may arise from π-π interactions between the enzyme and PCC domains or enzyme-enzyme interactions [60]. The atomic percentages are presented in Table 2, while the C/N ratios of covalently or non-covalently immobilized Tmbgl on PCC and PCCox are displayed in Table 3.

**Figure 7:**
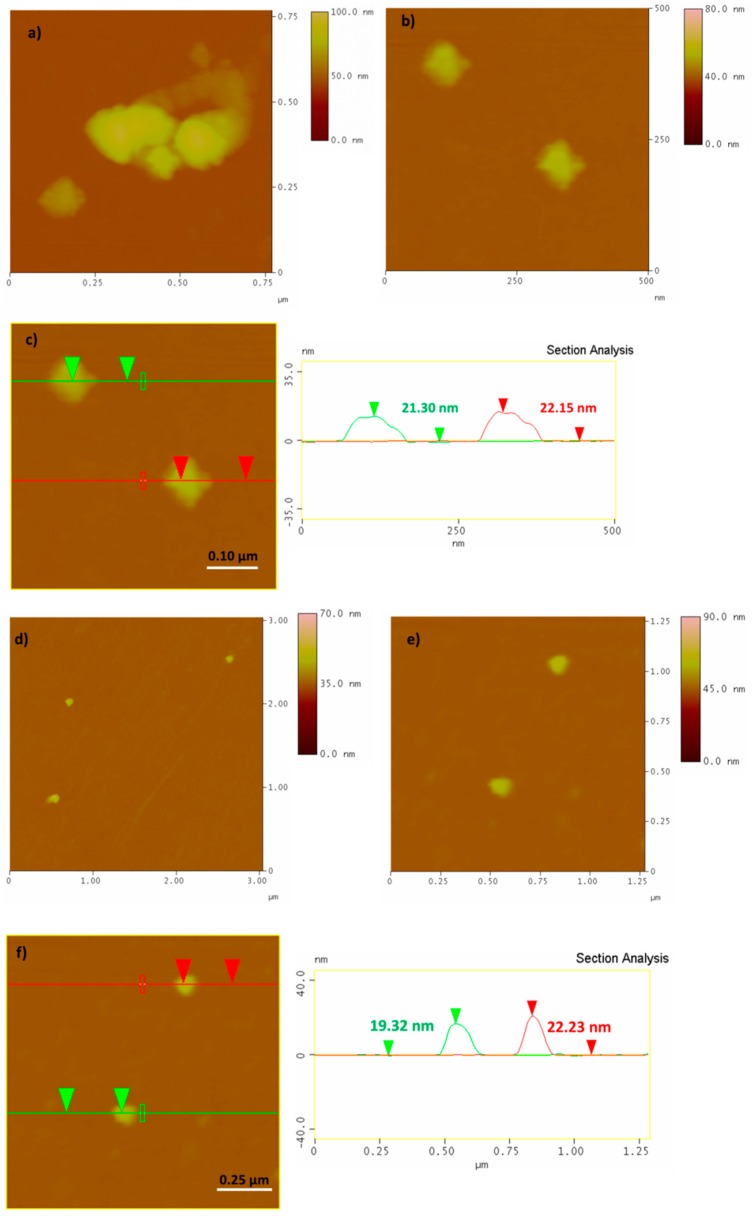
AFM height images and cross section analysis of PCC-Tmbgl (**a**–**c** images) and PCCox -Tmbgl (**d**–**f** images).

The immobilization of *β*-glucosidase on pristine and oxidized PCCs was confirmed via AFM (Figure 7). Representative AFM images indicate that molecules of *β*-glucosidase are attached on the surface of the PCC and PCCox. As revealed from the cross-section analysis, the size of the PCC is significantly increased, which verifies the successful attachment of the enzyme. The average thickness of PCC-Tmbgl-cov is 20 to 25 nm. In addition, the average thickness of PCCox-Tmbgl-cov is slightly decreased and has an average value of 18 to 23 nm. From these images, it is clear that the oxidation procedure affects the average size of the cuboids’ nanoparticles and also the estimated size of the nano-biocatalytic system.

### 3.4. Thermal Stability of Free and Immobilized β-Glucosidase

The thermal stability of free and immobilized *β*-glucosidases was investigated. The half-life time (the time required for the enzyme to lose 50% of its initial activity) was determined after incubation at 60 °C, in citrate phosphate buffer 100 mM, pH 5.0, and pH 6.5 for Albgl and Tmbgl, respectively. Table 4 demonstrates that the half-life time of covalently immobilized Albgl is enhanced by about 50%. These results indicate that the immobilized Albgl exhibits an increased resistance toward thermal denaturation that might be induced by heating. Similar enhanced thermal stability of immobilized *β*-glucosidase on various materials has been reported [35,61,62]. In the case of Tmbgl, both enzyme forms (free or immobilized) exhibited very high stability at 60 °C, as expected due to the well-known thermal stability of this enzyme. For instance, covalently immobilized Tmbgl on PCC or PCCox preserves more than 95% of its initial activity after incubation for 24 h at 60 °C.

### 3.5. Use of Immobilized β-Glucosidase for the Conversion of Oleuropein to Hydroxytyrosol

**Figure 8:**
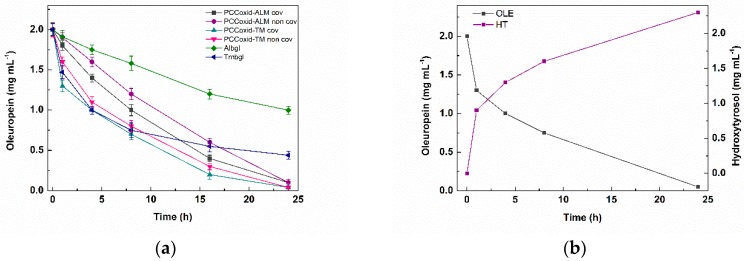
(**a**) Hydrolysis of OLE catalyzed by immobilized *β*-glucosidase at different time intervals. The amount of the enzyme in the reaction system was 0.15 mg/mL and 0.07 mg/mL for Albgl and Tmbgl respectively. (**b**) Reaction progress of the enzymatic hydrolysis of OLE catalyzed by PCCox-Tmbgl-cov and the formation of HT at different time intervals (the standard deviation was less than 5% in all cases).

Herein, free and immobilized *β*-glucosidases on PCCox were further used for the chemoenzymatic transformation of OLE to HT. More specific, as described in Figure 1, *β*-glucosidase catalyzes the hydrolysis of OLE to OLE aglycon (step 1), which, in aqueous solution at a pH of 7.0 and at 60 °C, it further undergoes a fast chemical re-arrangement, which leads to the formation of HT [23,63]. HPLC was used for the quantitative analysis of the reactants and products that were formed in the reaction mixture. The formation of the HT was also followed through liquid chromatography–mass spectrometry (LC-MS) analysis (Figures S1 and S2, Table S1, Supplementary Materials). Figure 8a illustrates the reaction progress of the enzymatic hydrolysis of OLE catalyzed by free and immobilized *β*-glucosidase. As can be seen in Table 5, free *β*-glucosidases demonstrate lower conversion rates than immobilized *β*-glucosidases, which could be attributed to the higher stability of the immobilized *β*-glucosidases compared to the free enzyme, in a similar manner as previously described (Table 4). It is worth noting that PCCox-Tmbgl preparations present higher hydrolytic activity than that observed for PCCox-Albgl, which is in accordance with that described in Table 5 for the hydrolysis of *p-*NPG. For all the tested bio-nanoconjugates, the OLE hydrolysis yield after 24 h of incubation exceeds 90%, which is higher than that reported for other *β*-glucosidase preparations under similar reaction conditions [27]. The progress of the formation of HT after the hydrolysis of OLE and the incubation of the reaction mixture at 60 °C at pH 7.0, is described in Figure 8b. The chemoenzymatic conversion of OLE that is described in the present work, results in the formation of 2.4 g L^−1^ of HT, which is among the highest reported concentrations in literature [27,29,64].

### 3.6. Reusability of Immobilized β-Glucosidase

**Figure 9:**
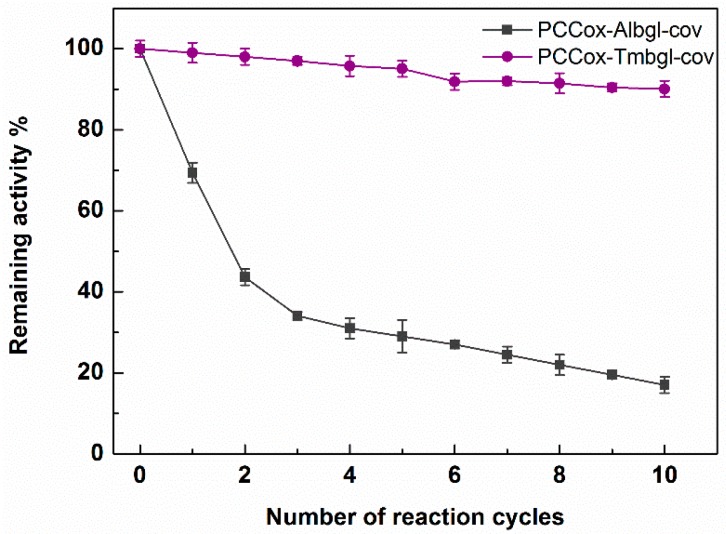
Reusability of covalently immobilized *β*-glucosidases on PCCox in citrate phosphate buffer 100 mM pH 5.0 and pH 6.5 for Albgl and Tmbgl, respectively, at 37 °C when the enzyme is covalently immobilized on PCCox.

Reuse of enzymes is pivotal for large-scale biocatalytic processes, especially from an economical point of view. In this work, we investigated the operational stability of PCCox-Albgl-cov and PCCox-Tmbgl-cov in multiple reaction cycles for the hydrolysis of OLE. Each reaction cycle was performed in citrate phosphate buffer 100 mM pH 5.0 and pH 6.5 for Albgl and Tmbgl, respectively, at 37 °C for 24 h. After each cycle, the biocatalyst was separated through centrifugation, washed with buffer, and reused. Figure 9 illustrates the remaining activity of immobilized *β*-glucosidases after each reaction cycle. After 10 cycles of reuse (240 h of total operation), the remaining activity of immobilized Albgl is reduced to 20%, which is similar to that observed when other nanomaterials were used as immobilization supports [35,49,62,65]. FTIR spectra analysis was employed in order to investigate possible conformational changes of the immobilized enzyme after the reuse process. The correlation coefficient r of FTIR spectra of PCCox-Albgl-cov before and after the reuse process is 0.94, which indicates that slight conformational changes might occur on the immobilized enzyme, which could explain the loss of its catalytic activity observed after repeated use. On the other hand, the remaining hydrolytic activity of immobilized Tmbgl is higher than 90% after 10 catalytic cycles. This indicates that PCCox-Tmbgl-cov bio-nanoconjugate is very stable and can be efficiently used for the biocatalytic conversion of natural compounds, such as OLE.

## 4. Conclusions

PCC and functionalized PCCox were used to develop novel nano-biocatalysts through non-covalent and covalent immobilization of *β*-glucosidases from two different organisms. The oxidation state of the nanomaterials that were used as immobilization supports and the immobilization procedure seem to affect the immobilization yield and the catalytic activity of the immobilized enzymes. The use of oxidized PCCs as a type of nano-support enhances the catalytic activity of the enzyme, which highlights the importance of the functionalization. The immobilized enzyme retains or exhibits higher thermal stability than the free enzyme, but this enhancement is not dependent on the oxidation state of the PCCs. The bio-nanoconjugates that are formed are able to efficiently catalyze the hydrolysis of OLE, which leads to the formation of a significant amount of HT. Immobilized *β*-Tmbgl on PCCox demonstrates high operational stability, which indicates—along with the excellent thermal stability- that this nano-biocatalyst is an efficient tool for the bioconversion of OLE and other natural compounds of industrial interest.

## Figures and Tables

**Table 1:** The immobilization yield (%) and enzymatic activity of *β*-glucosidase on PCC and PCCox after covalent and non-covalent immobilization (the standard deviation was less than 5% in all cases).

	Immobilization Yield %–(Activity U mg^−1^ Enzyme)			
	Albgl		Tmbgl	
	Covalent	Non-covalent	Covalent	Non-covalent
PCC	72–(0.8)	80–(0.5)	95–(24)	94–(18)
PCCox	90–(5)	62–(9)	93–(35)	94–(37)
free	9.5		45	

**Table 2:** Atomic percentages of PCC-Tmbgl-cov, PCC-Tmbgl-nc, PCCox-Tmbgl-cov, and PCCox-Tmbgl-nc.

Sample	Atomic Percentage %		
	C	O	N
PCC-Tmbgl-cov	75.1 ± 3.0	14.1 ± 1.1	10.8 ± 0.9
PCC-Tmbgl-nc	75.1 ± 3.0	13.5 ± 1.1	11.4 ± 0.9
PCCox-Tmbgl-cov	55.5 ± 2.2	40.7 ± 2.4	3.8 ± 0.3
PCCox-Tmbgl-nc	72 ± 2.9	23 ± 1.4	5 ± 0.4

**Table 3:** The C/N ratio of PCC-Tmbgl-cov, PCC-Tmbgl-nc, PCCox-Tmbgl-cov, and PCCox-Tmbgl-nc.

Sample	C/N Ratio
PCC-Tmbgl-cov	6.9
PCC-Tmbgl-nc	6.6
PCCox-Tmbgl-cov	14.6
PCCox-Tmbgl-nc	14.4

**Table 4:** Half-life time of free and immobilized Albgl on PCC and PCCox after incubation at 60 °C in citrate phosphate buffer 100 mM pH 5.0.

Sample	Half-Life Time (Hours)
Albgl	0.6 ± 0.04
PCC-Albgl-cov	0.9 ± 0.10
PCC-Albgl-nc	0.7 ± 0.07
PCCox-Albgl-cov	0.9 ± 0.06
PCCox-Albgl-nc	0.8 ± 0.05

**Table 5:** Initial reaction rates of immobilized *β*-glucosidase on PCC and PCCox for the hydrolysis of oleuropein (OLE) and percentage conversion yield of OLE after 24 h of incubation in citrate phosphate buffer 100 mM, pH 5.0, and pH 6.5 for Albgl and Tmbgl, respectively, at 37 °C (the standard deviation was less than 5% in all cases).

Sample	Initial Reaction Rate mM h^−1^ g^−1^ of Biocatalyst	% Conversion Yield of OLE
Free Albgl	0.05	50
PCCox-Albgl-cov	0.18	92
PCCox-Albgl-nc	0.16	90
Free Tmbgl	0.14	78
PCCox-Tmbgl-cov	0.20	98
PCCox-Tmbgl-nc	0.19	95

## Supplementary Materials

The following are available online at https://www.mdpi.com/2079-4991/9/8/1166/s1. Figure S1: HPLC chromatography of (a) OLE, (b) conversion of OLE step 1, and (c) conversion of OLE step 2, at 280 nm, Figure S2: (a) Total ion chromatogram (up) and UV chromatogram (down) at 280 nm of OLE (m/z 539) before the conversion. (b) Total ion chromatogram (up) and UV chromatogram (down) at 280 nm of the reaction mixture of the enzymatic conversion of OLE, step 1. (**c**) Total ion chromatogram (up) and UV chromatogram (down) at 280 nm of the reaction mixture of the conversion of OLE step 2, Table S1. Peak assignments of reaction mixture of conversion of OLE.
