# Supplementary material for: Enzymatic Conversion of Oleuropein to Hydroxytyrosol Using Immobilized β-Glucosidase on Porous Carbon Cuboids
Source: Nanomaterials (Basel). 2019 Aug 14;9(8):1166. doi: 10.3390/nano9081166 (PMC6724098; doi:10.3390/nano9081166)
Supplement: Supplementary file 1 [file nanomaterials-09-01166-s001.pdf]

nanomaterials

Enzymatic Conversion of Oleuropein to Hydroxytyrosol Using Immobilized *β*-Glucosidase on Porous Carbon Cuboids

Alexandra V. Chatzikonstantinou [1], Elena Gkantzou [1], Eleni Thomou [2], Nikolaos Chalmpes [2], Kyriaki-Marina Lyra [2], Vasiliki G. Kontogianni [3], Konstantinos Spyrou [2], Michaela Patila [1], Dimitrios Gournis [2,*] and Haralambos Stamatis [1,*]

[1] Biotechnology Laboratory, Department of Biological Applications and Technologies, University of Ioannina, Ioannina 45110, Greece
[2] Department of Materials Science & Engineering, University of Ioannina, Ioannina 45110, Greece
[3] Section of Organic Chemistry & Biochemistry, Department of Chemistry, University of Ioannina, Ioannina 45110, Greece
* Correspondence: dgourni@uoi.gr (D.G.); hstamati@uoi.gr (H.S.); Tel.: +30-2651007141; (D.G.); +30-2651007116 (H.S.)

1. HPLC analysis of OLE and HT during the conversion

Figure S1 illustrates the HPLC spectra of the different steps of the chemoenzymatic conversion of OLE to HT. The chromatogram of OLE before the conversion is depicted in Figure S1a. After the enzymatic hydrolysis of OLE from immobilized *β*-glucosidase for 24 h, the peak of OLE (20.19 min) is significantly reduced, while a number of wide peaks in the range of 20–23 min are observed (Figure S1b). These peaks could represent the intermediate products from OLE degradation[1]. A small peak of HT is also emerged at 7.7 min at this step. When the reaction mixture is incubated at 60 °C, pH 7 for 2 h, as Figure S1c shows (step 2) the peak of HT is significantly increased indicating the successful conversion of OLE to HT.

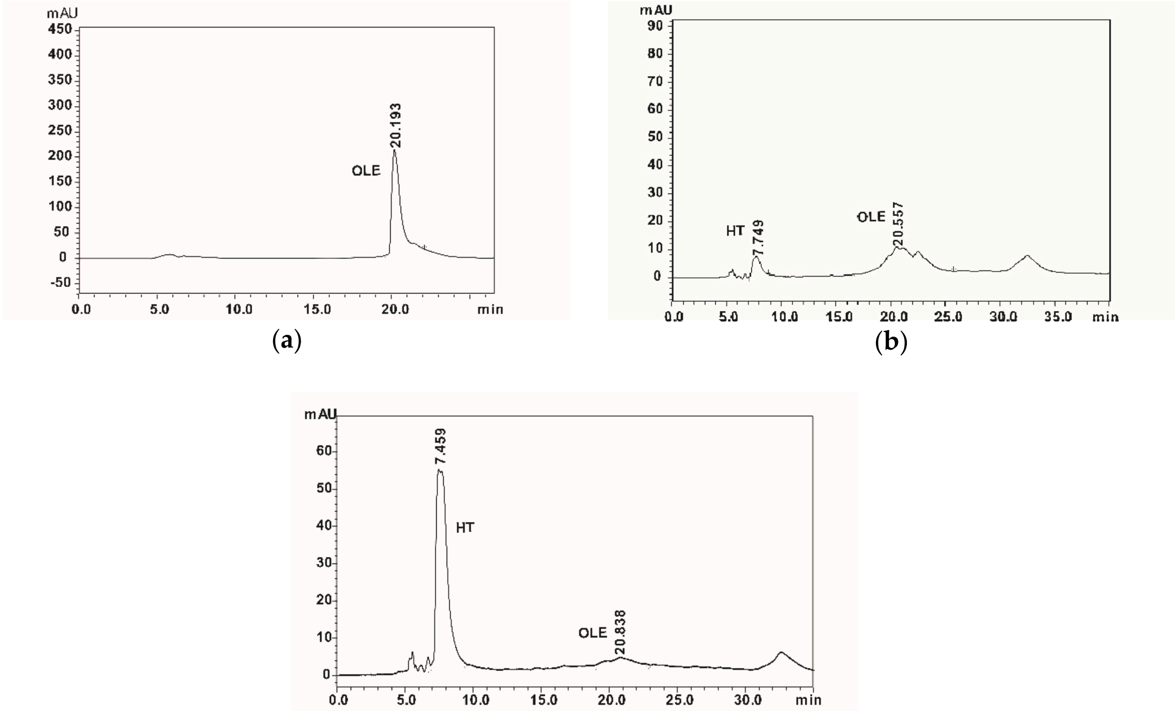

(a) (b)

(c)

Figure S1. HPLC chromatography of (**a**) OLE, (**b**) conversion of OLE step 1 and (**c**) conversion of OLE step 2, at 280 nm.

2. LC–MS analysis of the conversion of OLE to HT

2.1. Instrumentation

All LC-MS^n experiments were performed on a quadrupole ion trap mass analyzer (Agilent Technologies, model MSD trap SL) retrofitted to a 1100 binary HPLC system equipped with a degasser, an autosampler, a diode array detector and an electrospray ionization source (Agilent Technologies, Karlsruhe, Germany). All hardware components were controlled by Agilent Chemstation Software.

2.2. Analysis

A 10 μl aliquot was filtered (0.45 μm) and injected into the LC–MS instrument. Separation was achieved on a 15 cm x 4.6 mm i.d., 5 μm Zorbax Eclipse XDB-C18 analytical column (Agilent, USA), at a flow rate of 0.3 mL min^{-1} for oleuropein, using as solvent A (water/acetic acid, 99.9: 0.1 v/v) and solvent B (acetonitrile). The gradient used for the analysis was: 0–30 min, 80-50% A; 30–35 min 50% A; 35–40 min 50–80% A. The UV/vis spectra were recorded in the range of 200–550 nm and chromatograms were acquired at 254 and 280 nm.

Precursor ions scanning of chemoenzymatic conversion of OLE to HT was monitored between m/z 50–m/z 1.000 in negative polarity. The ionization source conditions were as follows: capillary voltage, 3.5 kV; drying gas temperature, 350 °C; nitrogen flow and pressure, 11 L min^{-1} and 50 psi, respectively. Maximum accumulation time of ion trap and the number of MS repetitions to obtain the MS average spectra were set at 30 and 3 ms, respectively.

2.3. Results of LC-MS analysis of the conversion of OLE

The chemoenzymatic conversion of OLE to HT was clarified with LC-MS experiments. The total ion chromatogram and the UV chromatogram at 280 nm of the conversion of OLE in each step is illustrated in Figure S2. Oleuropein is detected before the bioconversion at 17.4 min and corresponds to m/z 539 (Figure S2a). After the enzymatic hydrolysis of OLE from immobilized β-glucosidase for 24 h, the peak of OLE is significantly reduced while a number of peaks, peak 2 and peaks 4-9 are observed that correspond to m/z 377 and according to bibliography belong to different types of OLE-aglycones (Figure S1b)[2]. These data proves the successful hydrolysis of glucosidic bond of OLE from the immobilized β-glucosidase. A minor peak of HT is also detected at 6.9 min at this step, corresponding to m/z 123. After the incubation of the reaction mixture at 60 ^{o}C, pH 7 for 2 h (step 2) there is an increase of HT peak and a decrease of OLE-algycones peaks indicating the formation of HT during the step 2 of the conversion.

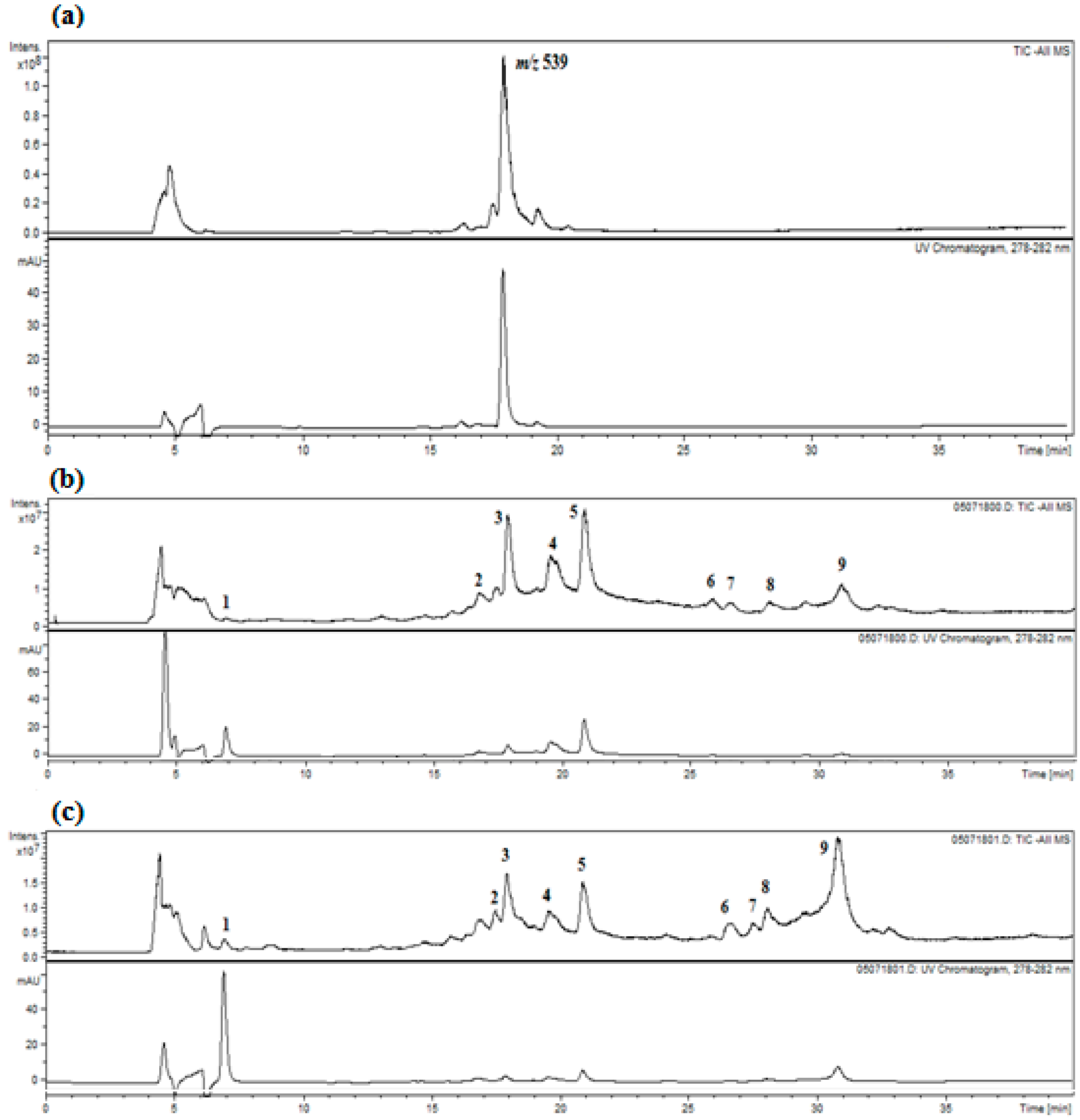

Figure S2. (**a**) Total ion chromatogram (up) and UV chromatogram (down) at 280 nm of OLE (m/z 539) before the conversion; (**b**) Total ion chromatogram (up) and UV chromatogram (down) at 280 nm of the reaction mixture of the enzymatic conversion of OLE, step 1; (**c**) Total ion chromatogram (up) and UV chromatogram (down) at 280 nm of the reaction mixture of the conversion of OLE step 2. See Table S1 for peak identification.

Table S1. Peak assignments of reaction mixture of conversionof OLE

**P.**	**Rt (min)**	**[M-H]- (m/z) (%)**	**Compounds**
1	6.9	123(100), 153(45)	HT
2	17.4	377(100), 713(56), 737(50), 307(35), 275(27), 349(22)	OLE-aglycon
3	17.9	539(100), 377(10)	OLE
4	19.5	377(100), 307(35), 275(28)	OLE-aglycon
5	20.9	377(100), 307(36), 275(29)	OLE-aglycon
6	26.6	377(100), 307(30), 275(25)	OLE-aglycon
7	27.5	377(100), 307(29), 275(23)	OLE-aglycon
8	28.0	377(100), 307(35), 275(25)	OLE-aglycon
9	30.8	377(100), 307(29), 275(21)	OLE-aglycon

References

1. Liu, M.; Yong, Q. Efficient bioconversion of oleuropein from olive leaf extract to antioxidant hydroxytyrosol by enzymatic hydrolysis and high-temperature degradation. *Biotechnol. Appl. Biochem.***2018**, *5*, 680–689.
2. Nikolaivits, E.; Termentzi, A.; Skaltsounis, A. Enzymatic tailoring of oleuropein from Olea europaea leaves and product identi fi cation by HRMS / MS spectrometry. *J. Biotechnol.***2017**, *253*, 48–54.

© 2019 by the authors. Submitted for possible open access publication under the terms and conditions of the Creative Commons Attribution (CC BY) license (http://creativecommons.org/licenses/by/4.0/).
